# Chimeric Antigen Receptor-Modified T Cell Immunotherapy for Relapsed and Refractory Adult Burkitt Lymphoma

**DOI:** 10.3389/fimmu.2022.879983

**Published:** 2022-05-20

**Authors:** Jiaying Wu, Yang Cao, Qi Zhang, Wanying Liu, Xiaoxi Zhou, Xi Ming, Fankai Meng, Yicheng Zhang, Chunrui Li, Liang Huang, Jia Wei, Miao Zheng, Shangkun Zhang, Tongcun Zhang, Xiaojian Zhu, Na Wang, Jue Wang, Gaoxiang Wang, Jianfeng Zhou, Bo Liu, Yi Xiao

**Affiliations:** ^1^ Department of Hematology, Tongji Hospital, Tongji Medical College, Huazhong University of Science and Technology, Wuhan, China; ^2^ Wuhan Bio-Raid Biotechnology Co. Ltd., Wuhan, China; ^3^ Department of Oncology, Tongji Hospital, Tongji Medical College, Huazhong University of Science and Technology, Wuhan, China

**Keywords:** Burkitt lymphoma, chimeric antigen receptor, immunotherapy, transplantation, cytokine release syndrome, immune effector cell-associated neurotoxicity syndrome

## Abstract

Patients with Burkitt lymphoma who are refractory to initial therapy or who relapse after undergoing intensive chemotherapy and autologous stem cell transplantation (ASCT) usually have a poor prognosis. While there has been considerable progress in the use of chimeric antigen receptor-modified (CAR) T cell immunotherapy for the treatment of relapsed and refractory (r/r) malignancies, explicit data on adult patients with r/r Burkitt lymphoma are limited. We conducted two single-arm clinical trials to evaluate the clinical efficacy and toxicity of CD19/CD22 CAR T cell immunotherapy both alone (trial A) and in combination with ASCT (trial B) in adult patients with r/r Burkitt lymphoma. In total, 28 adult patients with r/r Burkitt lymphoma were enrolled [trial A (n = 15) and trial B (n = 13)]. The median doses of CD22 and CD19 CAR T cell infusions were 4.1 × 10^6^/kg and 4.0 × 10^6^/kg, respectively. Subsequently, after CAR T cell infusion, overall and complete responses were observed in 19 (67.9%) and 16 (57.1%) patients, respectively. The cumulative incidence rates of grade 2–4 cytokine release syndrome and immune effector cell-associated neurotoxicity syndrome were 39.3% (11/28) and 10.7% (3/28), respectively. After a median follow-up duration of 12.5 months, 16 patients (5 in trial A and 11 in trial B) survived. Both the estimated 1-year progression-free and overall survival rates were 55.6%. Our preliminary results indicated that salvage therapy with CD19/CD22 CAR T cell infusion alone and that in combination with ASCT are effective in treating some adult patients with r/r Burkitt lymphoma.

## Introduction

Burkitt lymphoma is a rare and extremely aggressive subtype of B cell non-Hodgkin’s lymphoma (B-NHL). It is characterized by the dysregulation of the proto-oncogene *MYC*, which is commonly caused by a typical chromosomal translocation [t(8;14)] (1, 2). Burkitt lymphoma is highly sensitive to short-term intensive chemotherapy, and most patients can be treated using a multiagent regimen that includes rituximab (3). A large multicenter study including 30 cancer centers of the United States has shown that adult patients with Burkitt lymphoma have relatively favorable outcomes, with 3-year progression-free survival (PFS) and overall survival (OS) rates of 64% and 70%, respectively (4). However, relapsed and refractory (r/r) cases remain ineluctable, especially in patients with adverse prognostic factors or high-risk genetic abnormalities (5). Patients with Burkitt lymphoma who are refractory to initial therapy or who relapse after an intensive regimen and autologous stem cell transplantation (ASCT) usually have an extremely poor clinical prognosis (6, 7). Therefore, the early initiation of salvage therapies is essential for these patients owing to the invasive nature of the disease.

Treatment of patients with r/r Burkitt lymphoma *via* traditional or targeted chemotherapy produces poor outcomes. In a previous study, only two-fifths of patients with r/r Burkitt lymphoma or high-grade B cell lymphoma responded to second-line salvage chemotherapy, and even fewer patients achieved sustained remission (6). While ASCT shows therapeutic effects in some patients with r/r Burkitt lymphoma, chemotherapy-resistant patients obtain no significant benefit from further consolidative ASCT. Thus, combination with other innovative strategies may prove to be more effective for r/r high-risk subtypes (8). Recently, there has been considerable progress in the use of chimeric antigen receptor-modified (CAR) T cell immunotherapy for r/r malignancy treatment. Representative hematological diseases are r/r B cell acute lymphoblastic leukemia (B-ALL) and diffuse large B cell lymphoma (DLBCL) (9–11). Several case reports have demonstrated positive results for patients with r/r Burkitt lymphoma receiving CAR T cell immunotherapy (12–15); however, explicit data on CAR T cell immunotherapy alone or in combination with ASCT are still lacking.

In this study, we aimed to systematically evaluate the outcomes of adult patients with r/r Burkitt lymphoma treated with CAR T cell immunotherapy. We conducted two single-arm clinical trials to explore the clinical efficacy and related toxicity of CD19/CD22 CAR T cell immunotherapy alone and in combination with ASCT in adult patients with r/r Burkitt lymphoma.

## Methods

### Study Design

**Figure 1:**
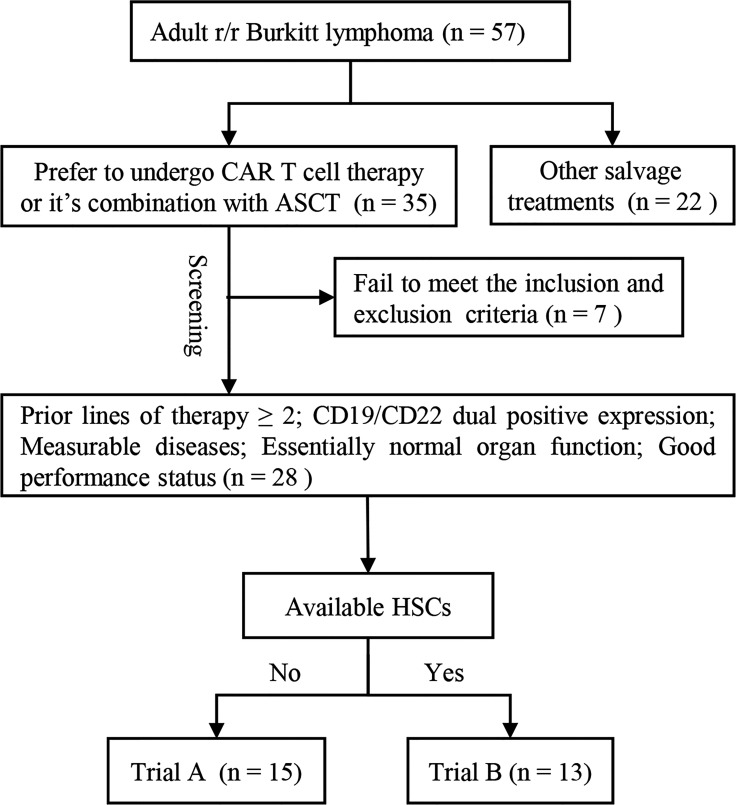
Participant screening flow chart. Adult patients with r/r Burkitt lymphoma who opted to undergo CAR T cell immunotherapy or CAR T cell immunotherapy combined with ASCT entered the screening progress. All participants were r/r after prior treatments (therapy lines ≥ 2). The positive expression of CD19 and CD22 was confirmed *via* flow cytometry or immunohistochemical analysis. Measurable diseases, normal organ function, and good performance status were necessary for inclusion. Patients with uncontrollable infections were excluded. Patients with enough autologous HSCs were included in trial B and those without enough autologous HSCs were included in trial A. ASCT, autologous stem cell transplantation; CAR, chimeric antigen receptor; HSC, hematopoietic stem cell; r/r, refractory/relapsed; trial A, CD19/CD22 CAR T cell immunotherapy; trial B, CD19/CD22 CAR T cell immunotherapy following ASCT.

According to the availability of hematopoietic stem cells (HSC) and patients’ choice, adult patients with r/r Burkitt lymphoma were enrolled in the screening process of our two single-arm clinical trials—treatment of r/r B cell malignancies with CD19/CD22 CAR T cell immunotherapy (trial A) or CD19/CD22 CAR T cell immunotherapy combined with ASCT (trial B). Patients with insufficient autologous HSCs were assigned to trial A, whereas those with sufficient HSCs were assigned to trial B. The screening flow chart is shown in **Figure 1**. All participants were r/r after prior treatments (therapy lines ≥ 2). The positive expression of CD19 and CD22 in tumor cells was confirmed *via* flow cytometry or immunohistochemical analysis. Inclusion criteria were the presence of measurable diseases, normal organ functions, and good performance status. Patients with uncontrollable infections were excluded. This study was conducted in accordance with the tenets of the 1964 Declaration of Helsinki and approved by the Ethics Committee of Tongji Hospital, Tongji Medical College, Huazhong University of Science and Technology. Patients and their families provided informed consent to study participation. Consent to publication was not applicable. The trials have been registered in the Chinese Clinical Trial Registry (https://www.chictr.org.cn/index.aspx; registration no.: ChiCTR-OPN-16008526 and ChiCTR-OPN-16009847).

### Therapeutic Procedures

#### Leukapheresis and Lymphodepletion

The therapy flow charts of the clinical trials were reported in previous studies (16, 17). In trial A, the participants received peripheral blood mononuclear cell (PBMC) apheresis for CAR T cell preparation. For lymphodepletion, fludarabine [25 mg/(m^2^·d)] and cyclophosphamide [300mg/(m^2^·d)] were administered for 3 consecutive days (days −4 to −2). CD22 and CD19 CAR T cells were separately infused on consecutive days starting from Day 0. In trial B, the participants underwent two separate apheresis procedures (granulocyte colony-stimulating factor-stimulated autologous HSC collection and PBMC apheresis). The standard BEAM conditioning regimen was given before autologous HSC infusion on day 0. It comprised the administration of 300 mg/(m^2^·d) of carmustine on Day −6, 200 mg/(m^2^·d) of etoposide on Days −5 to −2, 400 mg/(m^2^·d) of cytarabine on Days −5 to −2, and 140 mg/(m^2^·d) of melphalan on Day −1. CD22 and CD19 CAR T cells were separately infused within 2–6 days after autologous HSC infusion.

#### Lentivirus Construction

As described in a previous study conducted at our center (18), the third-generation CD19 or CD22 CAR lentiviral vectors used in our trials encode a single-chain fragment variable derived from a murine monoclonal antibody against human CD19 or CD22 linked with two costimulatory domains (CD28 and 4-1BB) and the CD3-zeta T cell activation domain. The manufacturing procedure and related quality control of CAR T cells were conducted by Wuhan Bio-Raid Biotechnology Co. Ltd.

### Efficacy and Toxicity Assessment

The primary endpoint was the evaluation of the objective response rates (ORR). This was based on the National Comprehensive Cancer Network guidelines and Lugano Response Criteria for B cell lymphoma (19). Cytokine release syndrome (CRS) and immune effector cell-associated neurotoxicity syndrome (ICANS) were evaluated and graded according to the American Society for Transplantation and Cellular Therapy Consensus Criteria (20, 21). Patients with severe toxicities received supportive care plus glucocorticoid and/or tocilizumab therapy. The expansion of CAR T cells *in vivo* was assessed using droplet digital polymerase chain reaction technology. PFS was defined as the time from the first CAR T cell infusion to progression, death, or final follow-up, and OS was defined as the time from the first CAR T cell infusion to death or final follow-up.

### Statistical Analysis

Continuous variables were presented as medians and ranges, and categorical variables as frequencies and percentages. An independent two-sample *t*-test or nonparametric test was used to assess continuous variables between groups, and the chi-square or Fisher’s exact test was used to evaluate categorical variables. The probability rates of PFS and OS were analyzed using the Kaplan–Meier method, and between-group comparisons were performed using log-rank tests. A two-tailed *p*-value of <0.05 indicated statistically significant differences. All statistical analyses were performed using the SPSS for Windows, v.26 (IBM Corp., Armonk, NY) and GraphPad Prism for Windows v. 8.0 (GraphPad Software, San Diego, CA).

## Results

### Characteristics of Patients and Treatments

Between January 1, 2016 and July 31, 2021, 28 adult patients [median age: 32 (17–70) years] with r/r Burkitt lymphoma were enrolled in two clinical trials conducted by our department—CD19/CD22 CAR T cell immunotherapy (trial A, n = 15) and CD19/CD22 CAR T cell immunotherapy combined with ASCT (trial B, n = 13). Upon diagnosis, 25 of the 28 patients (89.3%) had advanced-stage disease, and 20 (71.4%) patients had a high-risk subtype, with a Burkitt Lymphoma International Prognostic Index score of ≥2. High-risk genetic abnormalities included *TP53* mutations (15/28, 53.6%), *ID3* mutations (11/28, 39.3%), *DDX3X* mutations (10/28, 35.7%), and *MYC* mutations (11/28, 39.3%). All patients received standard short-course intensive chemotherapy with a median of 3 lines of prior therapy (range: 2 to 7 lines). Of the 28 patients, 21 (75.0%) were refractory to initial or multiline treatments, and the remaining 7 patients achieved complete response (CR) but relapsed. At the beginning of our study, 10 patients still had large lesions (≥7 cm). Baseline characteristics and subgroup data are shown in **Table 1**.

**Table 1:** The baseline and treatment characteristics of the adult patients with relapsed/refractory Burkitt lymphoma enrolled in our study.

Characteristics	Total (n = 28)	Subgroups		*P*-value^#^
		Trial A (n = 15)	Trial B (n = 13)	
Age (y), median (range)	32 (17-70)	30 (17-62)	33 (17-70)	0.26
Gender, n (%)				1.00
Men	16 (57.1)	9 (60.0)	7 (53.8)	
Women	12 (42.9)	6 (40.0)	6 (46.2)	
**At diagnosis**				
Stage*, n (%)				0.09
I-II	3 (10.7)	0 (0)	3 (23.1)	
III-IV	25 (89.3)	15 (100.0)	10 (76.9)	
BL-IPI score, n (%)				1.00
0-1	8 (28.6)	4 (26.7)	4 (30.8)	
2-4	20 (71.4)	11 (73.3)	9 (69.2)	
Genetics, n (%)				
* TP53* mutation	15 (53.6)	7 (46.7)	8 (61.5)	0.48
* ID3* mutation	11 (39.3)	3 (20.0)	8 (61.5)	0.05
* DDX3X* mutation	10 (35.7)	5 (33.3)	5 (38.5)	1.00
* c-MYC* mutation	11 (39.3)	5 (33.3)	6 (46.2)	0.70
**At study entry**				
Disease type, n (%)				0.40
Primary refractory	21 (75.0)	10 (66.7)	11 (84.6)	
Relapsed	7 (25.0)	5 (33.3)	2 (15.4)	
Prior lines of therapy, median (range)	3 (2-7)	3 (2-7)	3 (2-7)	0.26
ECOG PS, n (%)				0.13
0-1	14 (50.0)	5 (33.3)	9 (69.2)	
≥2	14 (50.0)	10 (66.7)	4 (30.8)	
LDH, median (range)	266 (105-1867)	444 (122-1867)	223 (105-437)	0.03
Bulky≥7.0cm, n (%)	10 (35.7)	8 (53.3)	2 (15.4)	0.06
Disease status, n (%)				0.04
PR	9 (32.1)	2 (13.3)	7 (53.8)	
SD/PD	19 (67.9)	13 (86.7)	6 (46.2)	
**Treatment**				
Infusion cells (× 10^6^/kg), median (range)				
CD22 CAR T cells	4.1 (1.6-8.3)	4.2 (2.0-8.3)	4.0 (1.6-6.7)	0.98
CD19 CAR T cells	4.0 (1.3-6.3)	4.0 (2.7-6.2)	4.0 (1.3-6.3)	0.61

Y, years; BL-IPI, Burkitt Lymphoma International Prognostic Index; CAR, chimeric antigen receptor; ECOG PS, Eastern Cooperative Oncology Group Performance Status; LDH, lactate dehydrogenase; PD, progression of disease; PR, partial response; SD, stable disease; trial A, CD19/CD22 CAR T cell immunotherapy; trial B, CD19/CD22 CAR T cell immunotherapy following autologous stem cell transplantation. *Ann Arbor stage. **^#^** The comparison between trial A and trial B, p-value < 0.5 was considered statistically significant.

The CD22 and CD19 CAR T cells were sequentially infused at a median dose of 4.1 (range: 1.6–8.3) × 10^6^/kg and 4.0 (range: 1.3–6.3) × 10^6^/kg, respectively. In trial B, a median of 2.97 (1.43–7.92) × 10^6^/kg CD34^+^ cells were infused. All participants underwent successful neutrophil engraftment within 28 days after HSC infusion. In five cases, delayed platelet engraftment was observed.

### Response and Survival

Of the 28 patients, 19 eventually responded to the CAR T cell therapy; these included 16 patients who achieved CR and 3 who achieved partial response (PR). The best ORR and CR rate (CRR) were 67.9% (19/28) and 57.1% (16/28), respectively. The three patients with PR status subsequently experienced uncontrollable disease progression and eventually died. However, none of the participants who achieved CR experienced recurrence during long-term follow-up. Furthermore, 10 of the 16 patients with CR status (62.5%) stayed in durable remission for >18 months. The ORRs of trial A and trial B were 46.7% (7/15) and 92.3% (12/13), respectively (*p* = 0.02), and the CRRs were 33.3% (5/15) and 84.6% (11/13), respectively (*p* = 0.009).

**Figure 2:**
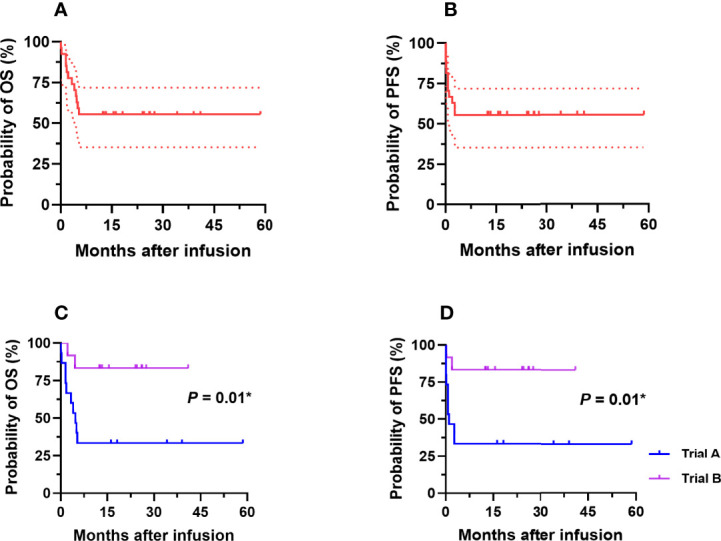
Probabilities of OS and PFS of adult patients with r/r Burkitt lymphoma enrolled in our study. **(A, B)** The estimated 1-year PFS and OS of all participants were 55.6% (95%CI: 35.2–71.8%) and 55.6% (95% CI: 35.2%–71.8%), respectively. **(C, D)** The estimated 1-year PFS rates of the patients in trial A and trial B were 33.3% (95% CI: 12.1%–56.4%) and 83.3% (95% CI: 48.2%–95.6%), respectively (*p* = 0.01), which were the same as the 1-year OS rates. OS, overall survival; PFS, progression-free survival; r/r, refractory/relapsed. *The comparison was considered statistically significant.

As of December 31, 2021, after a median follow-up duration of 12.5 (range: 0.1–58.6) months, 16 patients (5 in trial A, 11 in trial B) survived. The remaining 12 died due to rapid disease progression, with a median survival time of 1.87 (range: 0.1–5.3) months. Among these 12 patients, 9 did not respond and 3 partially responded to our therapy. The median PFS and OS of all participants were unreached, and the estimated 1-year PFS and OS rates were 55.6% (95% CI: 35.2%–71.8%) and 55.6% (95% CI: 35.2%–71.8%), respectively (**Figures 2A, B**). The median PFS and OS of the patients in trial A were 1.0 and 4.7 months, respectively, and the patients in trial B did not achieve median PFS and OS. The estimated 1-year PFS rate of the patients in trials A and B were 33.3% (95% CI: 12.1%–56.4%) and 83.3% (95% CI: 48.2%–95.6%), respectively (*p* = 0.01). The OS was the same as the PFS rate (**Figures 2C, D**).

### Toxicity

**Figure 3:**
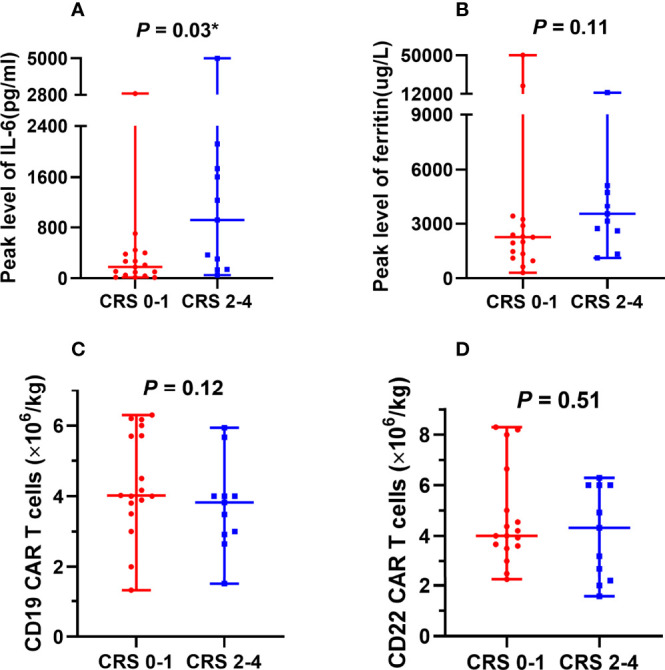
Factors associated with CRS. **(A)** Peak serum IL-6 levels were significantly higher in patients with grade 2–4 CRS than in patients with grade 0–1 CRS. **(B-D)** No significant differences were observed between serum ferritin levels, CAR T cell infusion doses, and CRS severity. CRS, cytokine release syndrome; IL-6, interleukin-6; CAR, chimeric antigen receptor. *The comparison was considered statistically significant.

After CAR T cell infusion, 27 of the 28 patients developed CRS. Additionally, 16 patients presented with fever (>38°C), which is grade 1 CRS, and 11 patients presented with hypotension and/or hypoxemia simultaneously, which is grade 2–4 CRS [grade 2, n = 5 (17.9%); grade 3–4, n = 6 (21.4%)]. One patient developed lethargy with an Immune Effector Cell-Associated Encephalopathy (ICE) score of 6 points (grade 2). One patient experienced seizure, with an ICE score of 4 points (grade 3). One patient was unarousable and ICE scoring could not be performed (grade 4). The cumulative incidences of grade 2–4 and 3–4 ICANS were 10.7% (3/28) and 7.1% (2/28), respectively. Participants with grade 2–4 CRS and ICANS were treated with broad-spectrum antibiotics and intravenous methylprednisolone. Four patients with severe CRS also received anti-interleukin-6 (IL-6) therapy. One patient died due to rapid tumor central infiltration and irreversible severe CRS/ICANS. In other patients, the symptoms completely resolved, and none had residual neurological dysfunctions. Six patients in trial A and five in trial B presented with grade 2–4 CRS, with cumulative incidences of 40.0% (6/15) and 38.5% (5/13) (*p* = 1.00), respectively. The inflammatory markers ferritin and IL-6 increased to varying degrees and reached median peak levels (ferritin: 2664.1 µg/L and IL-6: 269.7 pg/mL) at 8.5 (range: 1–23) days and 5.5 (range: 2–12) days after the first CAR T cell infusion, respectively. The peak serum IL-6 level was significantly higher in patients with grade 2–4 CRS than in those with grade 0–1 CRS (**Figure 3A**); however, serum ferritin levels and CD22 and CD19 CAR T cell infusion doses did not differ between patients with grade 2–4 CRS and those with 0–1 CRS (**Figures 3B–D**).

### CAR T Cell Kinetics

**Figure 4:**
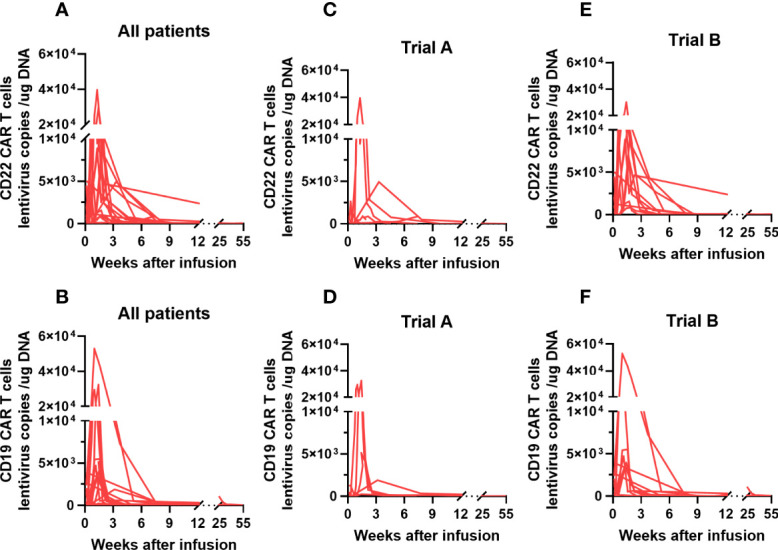
CAR T cell kinetics in adult patients with r/r Burkitt lymphoma enrolled in our study. **(A, B)** The median peak levels of CD22 and CD19 CAR T cell lentivirus copies in our study patients were 6951 (range: 141–39503) and 3930.5 (range: 125–69036) copies/µg DNA, respectively. **(C-F)** The median peak levels of CD22 and CD19 CAR T cell lentivirus copies were 2627 (range: 141–39503) and 1898 (range: 130–32422) copies/µg DNA, respectively, in trial A; and 9724 (range: 1000–30034) and 3939 (range: 125–69036) copies/µg DNA, respectively, in trial **(B)** CAR, chimeric antigen receptor; r/r, refractory/relapsed.

**Figure 5:**
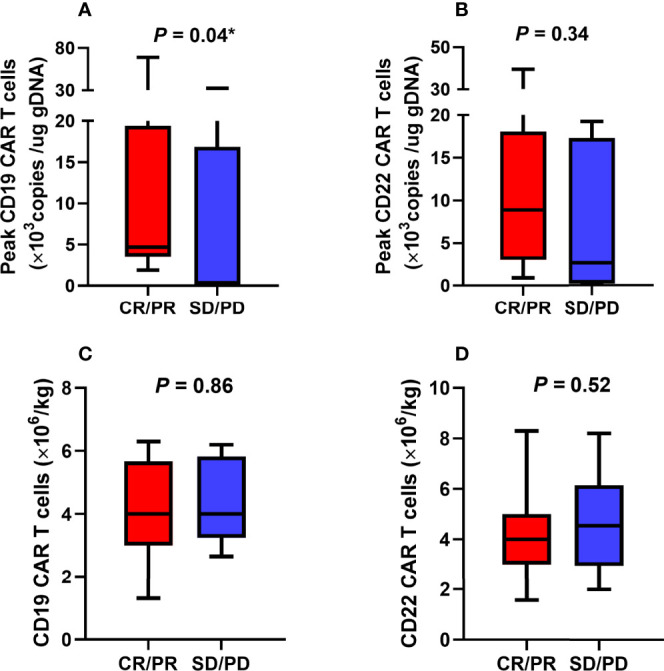
Association between CAR T cells and objective response. **(A)** The poor expansion of CD19 CAR T cells *in vivo* was associated with unsatisfactory efficacy. **(B-D)** There were no statistically significant correlations between CD22 CAR T cells expansion levels, CAR T cell infusion doses, and objective response. CAR, chimeric antigen receptor; CR, complete response; PD, progression of disease; PR, partial response; SD, stable disease. *The comparison was considered statistically significant.

The proliferation of CAR T cells *in vivo* was regularly monitored in 18 patients (trial A, n = 7; trial B, n = 11). The median peak lentivirus copy levels of CD22 and CD19 CAR T cells were 6951 (range: 141–39503) and 3930.5 (range: 125–69036) copies/µg DNA, respectively. The median times to peak levels were 1.07 (range: 0.14–3.29) and 1.14 (range: 0.14–3.14) weeks after CAR T cell infusion, respectively (**Figures 4A, B**). **Figures 4C–F** show the lentivirus copies in different trials, the median peak levels of CD22 and CD19 CAR T cell lentivirus copies were 2627 (range: 141–39503) and 1898 (range: 130–32422) copies/µg DNA, respectively, in trial A, whereas it was 9724 (range: 1000–30034) and 3939 (range: 125–69036) copies/µg DNA, respectively, in trial B. Although CAR T cell levels increased *in vivo*, the levels of CD19 CAR T cell lentivirus copies in patients who achieved objective response were higher than those in unresponsive patients (**Figure 5A**). Three months after infusion, only 4 of the 18 patients (22.2%) had consistently detectable levels of at least one transgene.

### Subgroup Analyses

As shown in **Table 2**, the participants’ objective response status differed according to the CAR T cell immunotherapy administered, Eastern Cooperative Oncology Group Performance Status (ECOG PS) scores, lactate dehydrogenase (LDH) levels, tumor size (<7.0 cm or ≥7.0 cm), and the presence of a *TP53* mutation. Poor rate of CD19 CAR T cell proliferation *in vivo* was associated with unsatisfactory efficacies (**Figure 5A**). However, there was no significant correlation between objective response and CD22 CAR T cell levels or between objective response and CD22 and CD19 CAR T cell infusion doses (**Figures 5B–D**).

**Table 2:** The best objective response rates of adult patients with refractory/relapsed Burkitt lymphoma according to baseline characteristics.

Subgroups	No. of patients	Objective response, n (%)	*P*-value
CAR T therapy option			0.02
Trial A	15	7 (46.7)	
Trial B	13	12 (92.3)	
Gender			1.00
Men	16	11 (68.8)	
Women	12	8 (66.7)	
Age (y)			0.11
≤30	12	6 (50.0)	
>30	16	13 (81.3)	
BL-IPI			1.00
0-1	8	6 (75.0)	
2-4	20	13 (65.0)	
*TP53* mutation			0.02
Yes	15	7 (46.7)	
No	13	12 (92.3)	
ECOG score			0.01
0-1	14	13 (92.9)	
2-4	14	6 (42.9)	
Prior therapy lines			0.21
≤4	18	14 (77.8)	
>4	10	5 (50.0)	
LDH level (u/l)			0.001
≤400	19	17 (89.5)	
>400	9	2 (22.2)	
Bulky ≥7.0 cm			<0.001
Yes	10	2 (20.0)	
No	18	17 (94.4)	
Disease status			0.20
PR	9	8 (88.9)	
SD/PD	19	11 (57.9)	

Y, years; BL-IPI, Burkitt Lymphoma International Prognostic Index; CAR, chimeric antigen receptor; ECOG PS, Eastern Cooperative Oncology Group Performance Status; LDH, lactate dehydrogenase; PD, progression of disease; PR, partial response; SD, stable disease; trial A, CD19/CD22 CAR T cell immunotherapy; trial B, CD19/CD22 CAR T cell immunotherapy following autologous stem cell transplantation.

## Discussion

CAR T cell immunotherapy can be beneficial to patients with r/r hematological tumors and r/r Burkitt lymphoma (13, 14). However, a systematic assessment has not been conducted previously. In the current study, adult patients with r/r Burkitt lymphoma receiving CD19/CD22 CAR T cell immunotherapy or its combination with ASCT achieved objective response. In total, 19 of the 28 adult patients (67.9%) with r/r Burkitt lymphoma included in our trials responded to the therapy. After a median follow-up of 12.5 months, 16 patients finally survived. The estimated 1-year PFS and OS rates were both 55.6%. Hence, our preliminary results suggest that some adult patients with r/r Burkitt lymphoma can benefit from novel treatment strategies involving CD19/CD22 CAR T cell immunotherapy or its combination with ASCT.

In trial A, 46.7% of the participants eventually achieved objective response, and 33.3% achieved long-term survival. Previously, in the ZUMA-1 study, 83.2% of patients with large B cell lymphoma achieved objective response to CD19 CAR T cell therapy (22). Additionally, in our previous study, we found the ORRs of patients with B-ALL and DLBCL treated with CD19/CD22 CAR T cell infusions in our department to be 96.0% and 72.2%, respectively (18). Thus, CAR T cell immunotherapy alone is thought to be less effective against r/r Burkitt lymphoma than other B cell malignancies in adults. Previous studies in the literature have speculated that this phenomenon might be caused by the tumor immunosuppressive microenvironment (TIME) and reduced antigen exposure, which impedes the effective proliferation of CAR T cells *in vivo* (23). The mechanism underlying the poor outcomes observed in adult patients with r/r Burkitt lymphoma should be further investigated. Recently, Liu et al. found that 65.4% of their pediatric r/r Burkitt lymphoma cases benefited from mouse-derived CD19 CAR T cell infusions; this rate is significantly higher than the rate observed in adult patients in the present study (24). Greater tumor homogeneity and different baseline characteristics could partially explain this discrepancy. That study also found that some pediatric patients who failed to respond or relapsed after receiving mouse-derived CD19 CAR T cell infusions subsequently responded to human-derived CAR T cell infusions; however, further research is required to determine whether this strategy is suitable for adults. Generally, CD19/CD22 CAR T cell infusions alone appear to be a viable treatment option for adult patients with r/r Burkitt lymphoma without enough HSCs, but the clinical efficacy was relatively limited.

Interestingly, in our study, CD19/CD22 CAR T cell infusions combined with ASCT appeared to lead to more favorable long-term outcomes in adult patients with r/r Burkitt lymphoma. The efficacy of this approach has previously been observed in patients with r/r B-NH and double-hit lymphoma (16, 25). A previous clinical trial conducted by our center reported an estimated 1-year PFS rate of 85.7% for patients with r/r B-NHL treated with CD19/CD22 CAR T cells and ASCT (25). For double-hit lymphoma, the estimated 2-year PFS rate reached 83.3% (16). In the current study, the estimated 1-year PFS rate for adult patients with r/r Burkitt lymphoma in trial B was 83.3%. This is roughly equivalent to the rates observed in the contemporary literature for other B cell hematological diseases. These robust results appear to be due to the synergistic effects of ASCT and CAR T cells. Relapse due to product contamination or residual disease is an urgent challenge in ASCT (26, 27). Targeted tumor-specific antigen-modified T cells can purify grafts and eradicate residual lesions after transplantation, effectively preventing recurrence. In addition, the standard BEAM conditioning regimen can considerably deplete lymphocytes and reduce tumor burden, and HSC infusion may modulate complex and heterogeneous TIME, enhancing the function and persistence of CAR T cells *in vivo* (17, 25, 28). For intermediate- or high-risk patients who fail to respond or relapse after initial therapy, HSC stimulation and CAR T cell therapy alongside the initiation of salvage chemoradiotherapy is recommended. This can guarantee a low tumor burden, thereby contributing to satisfactory curative effects (29, 30). However, whether this synergistic strategy is an appropriate second-line treatment option for adult patients with r/r Burkitt lymphoma remains to be confirmed. Meanwhile, further studies comparing ASCT in combination with CAR T cell immunotherapy with ASCT or CAR T cell immunotherapy alone should be conducted.

Subgroup analyses showed objective responses to be associated with ECOG PS score ≥ 2, LDH levels of >400 u/L, lesions ≥ 7.0 cm, the presence of a *TP53* mutation, and limited CD19 CAR T cell proliferation *in vivo*. Large lesions, which are common in Burkitt lymphoma, prevent CAR T cells from infiltrating tumors, fully contacting and recognizing cognate antigens, and performing their antitumor functions (23, 24). Besides, as shown in previous studies, the peak level of CAR T cell expansion is a significant predictor of clinical objective response (24, 31). Burkitt lymphoma has similarities to both leukemia and solid tumors. Therapeutic failure in some patients may be partly attributable to the limited increase in CAR T cell levels induced by complex and heterogeneous TIME (32). In our study, three partially responsive patients eventually experienced disease progression and died. Target antigen loss is an important mechanism of relapse or progression after CAR T cell immunotherapy (33, 34). Liu et al. assessed a pediatric patient with r/r Burkitt lymphoma who achieved CR status following mouse-derived CD19 CAR T cell infusion and eventually relapsed due to a loss of CD19 expression (24). Unfortunately, the three abovementioned patients did not undergo secondary biopsies at the time of progression.

CRS and ICANS are the two main toxicities observed in association with cellular therapy, and these were monitored and graded in this study. In a previous report on adult patients with r/r DLBCL who received tisagenlecleucel therapy, 22% experienced grade 3–4 CRS and 12% experienced neurological events (35). In a study on pediatric r/r Burkitt lymphoma cases, the incidence of grade 3–4 CRS and ICANS after treatment with mouse-derived CD19 CAR T cells were 34.8% and 24.1%, respectively (24). In another study, one of six (16.7%) adult patients with refractory Burkitt lymphomas was found to develop high-grade CRS after CD19/CD22 CAR T cell infusions (23). We observed no obvious differences in the incidence of severe CRS and ICANS compared to those previously reported. Our analysis also indicated that the levels of the major cytokine, IL-6, were significantly higher in patients with grades 2–4 CRS than in those with 0–1 CRS (36). However, the relatively lower incidence rate of grade 2–4 CRS seen in previous studies was not observed among patients in trial B of our study (17, 25). This might be because patients with r/r Burkitt lymphoma commonly present with large lesions and a high tumor burden and these factors are significantly associated with CRS severity after cellular therapy (36–38). Overall, adult patients with r/r Burkitt lymphoma were able to tolerate the toxicities associated with CAR T cell infusion.

To the best of our knowledge, this is the first systematic evaluation of CAR T cell immunotherapy outcomes in adult patients with r/r Burkitt lymphoma. We found that salvage therapy with CD19/CD22 CAR T cell infusions or CD19/CD22 CAR T cell infusions combined with ASCT is effective in some adult patients with r/r Burkitt lymphoma. Early initiation of CD19/CD22 CAR T cell therapy combined with ASCT appeared to be more beneficial. However, a randomized controlled cohort study is required to further validate our findings due to differences in some of the baseline characteristics of enrolled patients.

## Data Availability Statement

The raw data supporting the conclusions of this article will be made available by the authors, without undue reservation.

## Ethics Statement

The studies involving human participants were reviewed and approved by Chinese Clinical Trial Registry. The patients/participants provided their written informed consent to participate in this study.

## Author Contributions

JZ conceived and designed the study. YX revised the manuscript for submission. SZ and TZ supervised CAR T cell production and quality control. JiW and YC collected and analyzed data and wrote the paper. All authors conducted the clinical trials. All authors contributed to the article and approved the submitted version.

## Funding

This work was supported by the National Natural Science Foundation of China (grant numbers: 81873444 and 82070213).

## Conflict of Interest

SZ and TZ were employees of Wuhan Bio-Raid Biotechnology Co. Ltd.

The remaining authors declare that the research was conducted in the absence of any commercial or financial relationships that could be construed as a potential conflict of interest.

## Publisher’s Note

All claims expressed in this article are solely those of the authors and do not necessarily represent those of their affiliated organizations, or those of the publisher, the editors and the reviewers. Any product that may be evaluated in this article, or claim that may be made by its manufacturer, is not guaranteed or endorsed by the publisher.
